# Corticosteroids and Vertebral Trabecular Bone Quality in Women with Rheumatoid Arthritis

**DOI:** 10.3390/jcm14155223

**Published:** 2025-07-23

**Authors:** Jose Jorge Gomez-Camarena, Melissa Ramirez-Villafaña, Eli Efrain Gomez-Ramirez, Fabiola Gonzalez-Ponce, Miriam Fabiola Alcaraz-Lopez, Juan Manuel Ponce-Guarneros, Maria Luisa Vazquez-Villegas, Larissa Renne Rodriguez-Santillan, Norma Alejandra Rodriguez-Jimenez, Ana Miriam Saldaña-Cruz, Ernesto German Cardona-Muñoz, Sylvia Elena Totsuka-Sutto, Jorge Ivan Gamez-Nava, Laura Gonzalez-Lopez

**Affiliations:** 1 Programa de Doctorado en Farmacología, Instituto de Terapéutica Experimental y Clínica y Departamento de Fisiología, Centro Universitario de Ciencias de la Salud (CUCS), Universidad de Guadalajara, Guadalajara 44340, Mexico; jorgegomez59@gmail.com (J.J.G.-C.); melissa.ramirez@academicos.udg.mx (M.R.-V.); dr.efrain.gomez@gmail.com (E.E.G.-R.); fabiola.gonzalez@academicos.udg.mx (F.G.-P.); drponce85@gmail.com (J.M.P.-G.); larissarenne@gmail.com (L.R.R.-S.); norma.rodriguezj@academicos.udg.mx (N.A.R.-J.); ana.saldanac@academicos.udg.mx (A.M.S.-C.); cameg1@gmail.com (E.G.C.-M.); sytotsuka@gmail.com (S.E.T.-S.); 2 Departamento de Aparatos y Sistemas II, Universidad Autónoma de Guadalajara, Zapopan 45129, Mexico; 3 Departamento de Medicina Interna-Reumatología, Hospital General Regional 46, Instituto Mexicano del Seguro Social, Guadalajara 44910, Mexico; fabiola_alcaraz@hotmail.com; 4 Programa de Maestría, Departamento de Salud Publica, Centro Universitario de Ciencias de la Salud, Universidad de Guadalajara, Guadalajara 44340, Mexico; ma_luisavazquez@hotmail.com

**Keywords:** rheumatoid arthritis, trabecular bone score, glucocorticoids

## Abstract

**Background/Objectives:** Glucocorticoids (GCs) are frequently prescribed to control disease in Rheumatoid Arthritis (RA). However, long-term GC therapy with high daily doses is associated with bone involvement, which is considered the main extra-articular complication of RA. The trabecular bone score (TBS) has proven useful in assessing vertebral trabecular bone quality and fracture risk. To identify whether the long-term treatment of low doses of GCs are associated with low vertebral TBS in RA patients. **Methods:** A cross-sectional study, including 203 women with RA (ACR, 1987). Clinical, epidemiologic, and therapeutic variables were assessed. We identified the current daily dose, duration, and cumulative dose of GCs. Vertebral bone quality was assessed by TBS. Low vertebral trabecular bone quality was defined as TBS ≤ 1.300. Multivariate logistic regression analyses were used to identify risk factors of low TBS. **Results:** Prevalence of low TBS in RA women was 52%. RA + low TBS were older (61.9 vs. 55.5, *p* < 0.001) and had higher prevalence of menopause (90% vs. 75%, *p* = 0.004), hypertension (50% vs. 34%, *p* ≤ 0.02), and diabetes mellitus (13% vs. 4%, *p* = 0.02). There were no associations between GC use, neither doses or cumulative doses, and TBS. Multivariate analyses showed the following: age (OR: 1.05, 95% CI: 1.02–1.08) and the presence of diabetes mellitus (OR: 3.30, 95% CI: 1.03–10.60) were associated with a high risk of low vertebral trabecular bone quality in RA. **Conclusions:** Half of the RA patients had low trabecular bone quality. Older age and diabetes mellitus are important risk factors for low trabecular bone quality in RA. These findings should give alert to early detection of low TBS, establishing strategies aimed at avoiding the consequences of this complication, including vertebral fractures.

## 1. Introduction

Rheumatoid arthritis (RA) is the most frequently occurring chronic inflammatory joint disease, affecting around 1.6% of the Mexican population, with an age of onset between the fourth and fifth decades of life [1,2]. RA involves joint damage and systemic inflammation, with extra-articular manifestations including osteoporosis [3].

Osteoporosis in RA increases the risk of vertebral fragility fractures up to five times compared to the general population [4,5,6]. This high incidence of fractures is not totally explained by bone mineral density, and the bone quality of trabecular bone plays a relevant role [7].

Bone mineral density (BMD) constitutes a major parameter for identifying the risk of osteoporotic vertebral fractures; however, traditional BMD does not provide information regarding vertebral bone quality, which is related to the vertebral bone microarchitecture [8,9].

Trabecular bone score (TBS) is a textural index that evaluates pixel grey-level variations in the lumbar spine image using dual X-ray absorptiometry (DXA) [5]. TBS determination is a densitometry measurement of the bone microarchitecture, performed to indirectly assess the bone strength and is considered an important predictor of the risk of vertebral fractures [5,7].

Glucocorticoids (GCs) are frequently prescribed in pain management, stiffness, and swelling caused by RA [10]. However, long-term GC therapy is controverted because it is associated with decreased bone formation, increased bone resorption, and low BMD. The prevalence of glucocorticoid-induced osteoporosis has been described in 38% of patients with RA, and fractures were 13% [11]. Short-term treatment with GCs (<3 months) and in daily doses between 5 and 10 mg has been associated with a decrease in BMD in the lumbar spine and an increased risk of fracture [11].

Several studies have described an inverse relation between TBS score and glucocorticoid treatment for different populations, including Graves’ orbitopathy, asthma, menopausal women, and different autoimmune disease [12,13]. Likewise, it has been reported that TBS has greater discriminative power than BMD when used in the evaluation of fracture risk in patients with autoimmune disease treated with a chronic GC dose [12,13,14,15,16].

For a small group of patients with early RA, it has been described that, with the application of high doses of GCs, modifications in TBS occurred [17]. However, the use of high doses of GCs is uncommon in patients with RA, and most patients use doses lower than 7.5 mg. However, it is still unknown whether those patients treated with low doses of GCs over the long-term presented a decrease in trabecular bone quality with RA. The objective of this study was to identify whether the long-term treatment of low doses of GCs is associated with low vertebral TBS in RA patients.

## 2. Materials and Methods

### 2.1. Study Design: Cross-Sectional Study

#### 2.1.1. Study Population

All the patients were recruited from 15 February 2022 to 15 February 2023; we assessed 203 adult women with RA from an outpatient research clinic at a university center (Instituto de Terapéutica Experimental y Clínica, Centro Universitario de Ciencias de la Salud, Universidad de Guadalajara) in Guadalajara, Mexico.

#### 2.1.2. Inclusion and Exclusion Criteria

RA patients were eligible if they were women, aged ≥18 years, and fulfilled the 1987 American College of Rheumatology (ACR) classification criteria [18]. We excluded patients with overlapping syndrome, active infectious diseases including hepatitis B or C, human immunodeficiency virus or tuberculosis, hypo- or hyperthyroidism, hyperparathyroidism, chronic renal disease stages 4 or 5, cancer, or a record of treatment with osteo-formers or antiresorptive drugs.

#### 2.1.3. Ethics

This project was designed in accordance with the updated 1964 Helsinki Declaration from 2013 in Fortaleza, Brasil. This study was approved by the Research and Ethics Committee of the Instituto de Terapeutica Experimental y Clinica, Centro Universitario de Ciencias de la Salud, Universidad de Guadalajara. Code of approval: CI-00422. All the patients were invited to participate and signed a voluntary informed consent form before their inclusion.

#### 2.1.4. Study Protocol

Two trained researchers performed a clinical interview focused on epidemiological characteristics, comorbidities, clinical features, treatments, and the risk factors of osteoporosis and low vertebral trabecular bone quality.

#### 2.1.5. Epidemiological Variables

We included age, antecedents of smoking, alcohol consumption, menopause status, history of low-impact trauma fractures, height, weight, body mass Index (BMI), reduced physical activity, and comorbid diseases (diabetes mellitus and hypertension).

#### 2.1.6. Clinical Assessment

Disease activity was evaluated with the disease activity score of 28 joints using the erythrocyte sedimentation rate (DAS28-ESR) [19]. Functional disability was assessed with the Spanish version of the Health Assessment Questionnaire–Disability Index (HAQ-DI) [20].

Treatments with oral corticosteroids and anti-rheumatic drugs.

We assessed the long-term utilization of oral corticosteroids (prednisone, Deflazacort). We identified daily dose, duration, and cumulative dose expressed as prednisone equivalent. Low-dose was equal to or less than 7.5 mg/day. Long-term use of prednisone was defined as greater than 6 months.

We also investigated the use of synthetic and biological disease-modifying antirheumatic drugs (DMARDs).

#### 2.1.7. Bone Mineral Density Measurements

BMD was measured by DXA scan using a General Electric Lunar iDXA (Madison, WI, USA). All DXA scans were performed according to guidelines described by the International Society of Clinical Densitometry [21].

For BMD, we assessed two regions: (a) the anterior–posterior (AP) of lumbar region (L1–L4) and (b) the femoral neck. Osteoporosis was defined according to the ISCD criteria (International Society for Clinical Densitometry) where BMD is a T-Score −2.5 SD or more below that of a young, healthy adult; osteopenia is defined as a T-score between <−1 and −2.5 SD, and normal BMD as a T-score above −1.0 SD. This definition was applicable for patients ≥50 years of age or postmenopausal. Otherwise, for premenopausal women or an age lower than 50 years, a Z-score below −3 SD was considered as osteoporosis or low BMD in women with an age lower than 50 years. A Z-score of −2.0 or lower is defined as osteopenia or “below the expected range for age”, while a Z-score above −2.0 SD indicates normal BMD or “within the expected range for age” [21].

#### 2.1.8. Vertebral Bone Quality Assessment

We identified Lumbar TBS using TBS iNsight software version 1.8.1.0 (Medimaps group USA LLC, Wilmington, DE, USA). That measurement was obtained after analysis of the lumbar spine by DXA, with the index calculated as the mean value of the individual measurements for vertebral L1–L4.

According to their TBS results, RA patients were classified into two groups: (a) RA with low vertebral trabecular bone quality (TBS equal to or below 1.300); (b) RA with normal vertebral trabecular bone quality (TBS > 1.300) [22].

### 2.2. Statistical Analysis

Quantitative variables were expressed as means and standard deviations (SDs) and qualitative variables as frequencies and percentages (%). We compared proportions between groups [(a) low vertebral trabecular bone quality; (b) normal vertebral trabecular bone quality] using the Chi-square test or Fisher exact test. Comparisons between means were computed using Student’s *t* test.

We performed a multivariable logistic regression analysis to identify variables associated with the dependent variable (low bone trabecular quality, or TBS ≤ 1.300). The adjusted covariates introduced into the model were variables considered with biological plausibility or those variables in the univariable analysis with a significance ≤ 0.2. We included as covariates the age (years), presence of menopause and diabetes mellitus, body mass index (Kg/m^2^), disease duration (years), presence of functional disability, disease activity, and corticosteroid use. We utilized the enter and the forward stepwise methods. Statistical significance was set at the *p* ≤ 0.05 level. All statistical analyses were performed using R statistical software version 4.0.2.

## 3. Results

Table 1 shows the following characteristics: epidemiological, clinical, bone mineral density, and trabecular bone quality in women with RA. The mean age was 58.9 years, and most of them had a long-disease duration. Regarding their BMD results, 38% of patients were classified with osteoporosis, 36% with osteopenia, and 26% as normal densitometry. The mean of TBS L1-L4 was 1.301, classified with low vertebral trabecular bone quality in 52% of RA patients. Regarding therapeutic characteristics, 97% of RA patients used synthetic DMARDs, 10.3% biologic DMARDs, and 72% used corticosteroids (average dose of 4.3 mg/day and 5.8 years of use).

**Figure 1:**
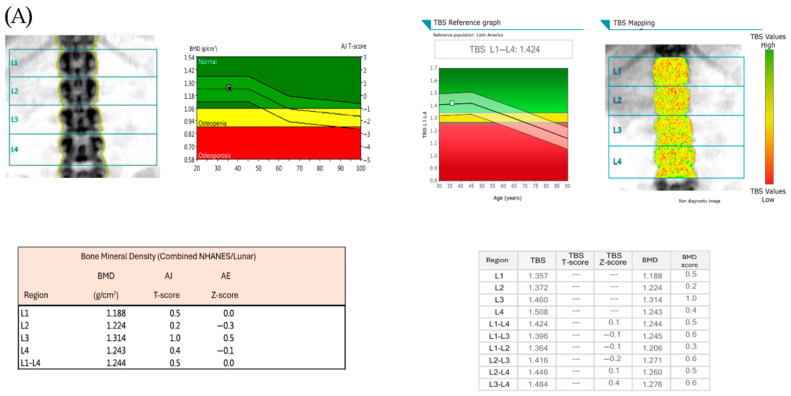

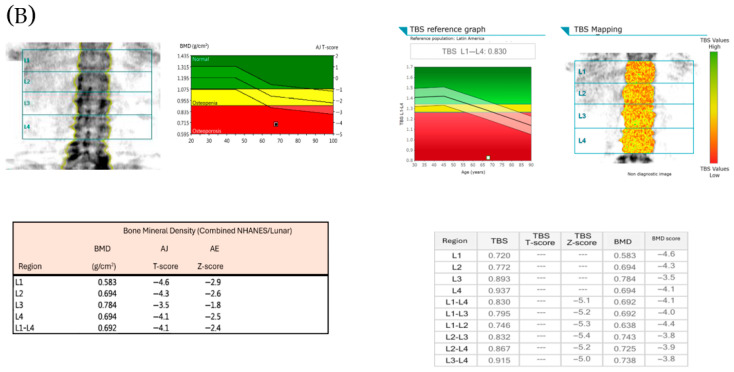
(**A**) shows images of bone mineral density by DXA and TBS measurements in one patient with normal BMD and normal TBS score (TBS > 1.300), and the individual BMD and TBS scores of the lumbar vertebrae (L1–L4). (**B**) shows images in one patient with osteoporosis and low TBS score (TBS ≤1.300), and the individual BMD and TBS scores of the lumbar vertebrae (L1–L4).

Figure 1A shows images of bone mineral density by DXA and TBS measurements in one patient with normal BMD and normal TBS score. Figure 1B shows images in one patient with osteoporosis and low TBS score.

**Figure 2:**
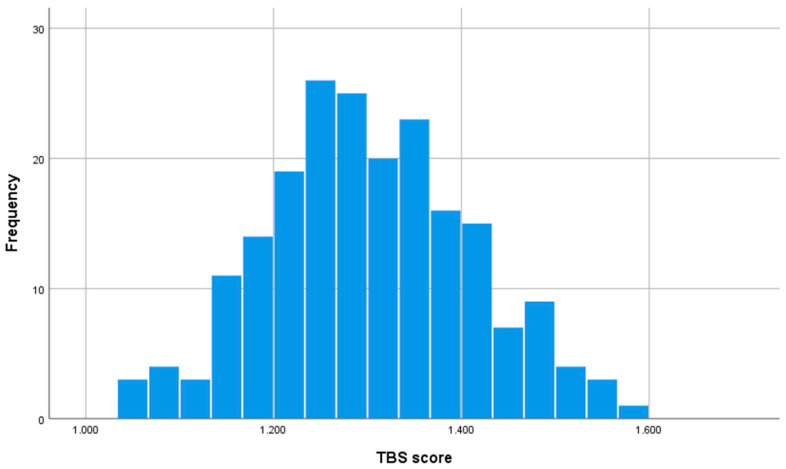
Frequency histogram showing the distribution of TBS scores of the patients included in the study. The height of each bar represents the number of patients with the TBS score. The values of the histogram are Mean TBS score: 1.301, standard deviation: 0.108, median: 1.295, range: 1.036–1.586.

Figure 2 shows the frequency histogram of TBS scores of the patients included in the study.

Table 2 compares the epidemiological and clinical characteristics in RA patients with normal and low vertebral TBS. RA patients with low vertebral TBS were older (61.9 vs. 55.5, *p* < 0.001) and had a higher prevalence of menopause (90% vs. 75%, *p* = 0.004), hypertension (50% vs. 34%, *p* ≤ 0.02), and diabetes mellitus (13% vs. 4%, *p* = 0.02) compared with RA + normal vertebral TBS. Likewise, the group of RA patients with low vertebral TBS presented a higher prevalence of osteoporosis (52% vs. 22%, *p* < 0.001). There were no differences in disease duration, severity of disease activity, functional disability, or treatment characteristics between groups.

Regarding the use of glucocorticoids, there were no differences observed in RA patients with low vertebral TBS vs. RA with normal vertebral TBS in frequency of use of corticosteroids (70% vs. 75%, respectively; *p* = 0.38), daily corticosteroid doses (4.0 vs. 4.6 mg/day, respectively; *p* = 0.15), time of using corticosteroids (5.4 vs. 6.4 years, respectively; *p* = 0.30), and cumulative doses of corticosteroids (31.2 vs. 40.4 g, respectively; *p* = 0.19).

Table 3 shows the results of the logistic regression analysis, assessing the variables associated with low vertebral trabecular bone quality in RA patients, after performing an adjustment by age, disease duration, corticosteroid use, disease activity, functional disability, and comorbidities. Using the stepwise method, after controlling for these potential confounders, two variables increased the risk of low vertebral trabecular bone quality in RA: age (OR: 1.05, 95% CI 1.02–1.08) and presence of diabetes mellitus (OR: 3.30, 95% CI 1.03–10.60).

## 4. Discussion

The present study showed that around half of RA patients have low vertebral trabecular bone quality, and 7 out of 10 patients with RA use corticosteroids. In our study was no association between using low-doses of GCs, time of using these drugs, or cumulative doses of GCs and the presence of low vertebral trabecular bone quality. Instead, the presence of diabetes mellitus and older age significantly increased the risk of low vertebral trabecular quality in RA patients in the multivariable analysis.

Nowadays it is clear that to initiate an early and tailored treatment in patients with RA and other rheumatic disorders constitutes a main determinant to avoid future sequels and complications, including osteoporosis and fractures. Recommendations by Clinical Practice Guidelines include the onset of MTX or other cs-DMARDs as early as the diagnosis of RA was achieved and when these drugs are insufficient for controlling the disease activity to initiate biologics or small-molecules [23,24]. The early onset of biologics is a recommendation for a subgroup of RA patients with more severe disease as well as other criteria of a worse prognosis, similar to other inflammatory disorders such as psoriasis or psoriatic arthritis [25]. However, in many countries the use of biologic agents is still limited in the majority of the patients due to economic restrictions. Therefore, the use of GCs is recommended by the treatment guidelines mainly as bridge therapy and considering a rapid discontinuation of these drugs [23]. Nevertheless, a high proportion of patients remain using GCs for months or even years, although most of them are using low doses of these drugs [26,27].

In this study we identified that 147/203 (72%) of our patients used corticosteroids, and 52% had low trabecular bone quality, using a TBS of ≤ 1.300 as the cut-off. These results agree with those found by Tavassoli et al. [28] in Iranian patients with RA who reported a prevalence of low TBS of 48.7% in their RA patients (using a cut-off of < 1.250). However, other authors have reported a higher frequency of low TBS in RA. Buehrinh et al. [29] identified a very high prevalence of low vertebral trabecular bone quality (87.4%) in German patients with RA (using a cut-off for TBS of < 1.310); however, the main age of their patients was significantly higher (mean 72 years) compared with our patients (mean age of 58.9 years).

Several studies have demonstrated that GCs can weaken bone microarchitecture in several ways, including hormonal dysregulation and the disturbance of bone metabolism, through mechanisms that involve an increase in osteoclast formation and the apoptosis of osteoblasts and osteocytes.

Regarding the use of corticosteroids, we observed that 72% of patients with RA use GCs, with an average dose of 4.3 mg/day. We did not observe an association between the use of corticosteroids, dose per day, cumulative dose and time of GC use, and the presence of low trabecular bone quality in RA.

These results agree with those of Koumakis et al. [30], who did not observe an association between corticosteroid use and TBS in French patients with RA. However, other authors have found different results; Corrado et al. [17] reported in Italian patients with early RA that exposure to corticosteroids for 12 months was associated with a decreasing mean of TBS. There were also other differences with our study, including the use of higher doses of GCs (mean doses of 9.5 mg/day in that study compared with 4.3 mg/day in our study) [14]. Kim D et al. [31] reported a negative correlation between TBS and dose of corticosteroids in their Korean patients with RA, although in that study a cut-off for defining a low vertebral TBS was not identified. Omichi Y et al. [32] reported that, among postmenopausal women with RA in Japan, the TBS was lower in patients receiving glucocorticoids (mean dose: 2.7 ± 1.3 mg/day) compared to those not receiving glucocorticoids [32]. Finally, in French patients with RA [33], it was observed by Bréban, et al. [33] that the TBS score was lower in users of corticosteroids compared to patients that were nonusers. However, these authors did not perform an adjusted analysis to identify if the low vertebral quality was truly associated with corticosteroids or explained by confounders, and neither assessed the cumulative doses a relevant characteristic of GC use in terms of potential influence on trabecular bone. Therefore, we consider that our study provides a more comprehensive statistical analysis of several factors potentially associated with low quality of vertebral trabecular bone. In this sense, our results do not show an association between TBS and GCs, probably because our patients used lower doses. Therefore, these confounding factors can influence the presence of degraded vertebral bone quality in RA patients.

Regarding other clinical characteristics in RA patients, we have not observed an association between low TBS and disease activity, functional disability, or inflammatory markers. These results agree with those described by Kim D et al. [31] in a study performed in Korea, where there was no correlation observed between TBS and disease activity score or erythrocyte sedimentation rate in their RA patients. Contrary to these results, Senosi et al. [34] reported a correlation between TBS score and longer disease duration, a higher mHAQ score and elevated Anti-CCP levels in Tunisian RA patients. Ioniță-Radu et al. [35] reported in a retrospective study that TBS score can be influenced by the response to cs-DMARDs, biologic agents, vitamin D levels, and high homocysteine levels. Ruaro et al. [36] review some studies assessing TBS scores performed in RA. These studies show that patients have lower values of TBS score compared to controls. Given the value of TBS score as an independent risk factor for osteoporotic fractures, the evidence of studies assessing the variables associated with low TBS in RA patients is mandatory.

We identified that a low TBS score in RA is highly prevalent in RA and can be influenced by comorbid diseases such as diabetes mellitus; but interestingly, low doses of corticosteroids seem not be a clear determinant for the low TBS values observed in these patients.

Similarly to what was observed by us, Wiebe et al. identified in a large sample of patients with different rheumatic disorders including rheumatoid arthritis that low doses of GCs ≤ 5 mg/day are not associated with a reduction of BMD in patients with inflammatory rheumatic diseases due to the effects of these drugs on inflammation [37].

The generalized deterioration of bone microarchitecture often occurs in patients with RA and is related not only to specific disease factors such as systemic inflammation, disease duration, and treatment, but also to primary factors (older age, female gender, low body mass index, reduced mobility, diabetes mellitus, etc.). These factors are as much relevant in RA as the variables associated with the disease or treatments.

Our findings are consistent with those reports in the literature; after controlling for confounders in the multivariate logistic regression analysis, we identified that the main risk factors associated with low trabecular bone quality in RA patients were greater age and diabetes mellitus. These epidemiological factors should be considered when assessing TBS scores in any population [37,38,39]. We observed that type 2 diabetes can be considered in the list of factors determining a deteriorated vertebral trabecular quality. Although the mechanism linking TBS and type 2 diabetes remains unclear, patients with type 2 diabetes have been reported to have lower TBS than non-diabetic individuals, suggesting its potential utility in assessing fracture risk in this population [36,38].

To the best of our knowledge, there is still little information available to identify the main factors associated with alterations of vertebral trabecular bone quality in women with RA. The present study explores multiple factors and includes the possible relationship between low-dose glucocorticoid use and altered trabecular bone quality. This study shows that low-dose glucocorticoids were not a determinant in explaining the high frequency of alterations in trabecular bone quality in these patients.

Our study evaluated the relations of TBS and corticosteroid use in women with RA, analyzed through the adjustment of possible confounding factors, and the interactions between low trabecular bone quality and other known clinical risk factors in these patients, which constitutes an additional strength of our study.

Likewise, another strength of our study is greater sample size, which allowed us to evaluate a broader range of risk factors associated with low vertebral bone quality in our RA patients through multivariate analysis compared to previously reported studies. Although our study is limited because it is cross-sectional and it is not possible to identify causal associations, we also cannot identify the time at which patients began to show a decrease in the quality of their vertebral bone.

Additional limitations of our study include that we did not measure other potential risk factors associated with the disease, such as elevated levels of cytokines like TNF-α and IL-6, or the measurement of blood levels of calcium or vitamin D, which are linked to the deterioration of vertebral trabecular bone quality. Finally, this study included only women with RA; therefore, the results cannot be extrapolated to men.

TBS is considered an important independent predictor of vertebral fracture risk over standard BMD assessment [14,15,16], so the inclusion of this TBS measure allows for the better planning of treatment strategies and the prevention of vertebral fractures.

## 5. Conclusions

In conclusion, in this study, half of the RA patients had low trabecular bone quality; older age and diabetes mellitus are important risk factors for low trabecular bone quality in RA, independent of traditional risk factors. These findings should alert clinicians to the need for early detection of low trabecular bone quality in RA patients, establishing strategies aimed at avoiding the consequences of this complication, including vertebral fractures. Despite the fact that 7 out of 10 RA patients used ≤5 mg/day of prednisone or equivalent in this study, these low-doses of glucocorticoids were not associated with poor trabecular bone quality. Further long-term studies and follow-up are required to identify the rate of progression to bone quality loss, thus helping us to reduce the impact on these RA patients. Therefore, periodic measurements of vertebral trabecular bone quality are required to identify its role as a predictor of vertebral fracture.

## Figures and Tables

**Table 1:** Characteristics: epidemiological, clinical, bone mineral density, and trabecular bone quality in Mexican women with rheumatoid arthritis.

Variable	*n* = 203
Age in years: mean ± SD	58.9 ± 11.4
Menopause, *n* (%)	169 (83)
Sedentary lifestyle, *n* (%)	136 (67)
Smoking, *n* (%)	21 (10)
History of fragility fractures, *n* (%)	17 (5.8)
Diabetes Mellitus type 2, *n* (%)	18 (9)
Hypertension, *n* (%)	86 (42)
Overweight/obesity, *n* (%)	145 (71)
*RA characteristics*	
Years of duration of RA: mean ± SD	13.6 ± 9.7
Functional disability (HAQ-DI > 0.6), *n* (%)	86 (42)
Disease activity (DAS28-ESR ≥ 2.6), *n* (%)	144 (71)
*Densitometry variables*	
Bone mineral density (BMD), gr/cm^2^: mean ± SD	1.8 ± 0.3
Central DXA results	
Normal BMD, *n* (%)	52 (26)
Osteopenia, *n* (%)	74 (36)
Osteoporosis, *n* (%)	77 (38)
*Trabecular Bone quality*	
TBS L1–L4, index: mean ± SD	1.301 ± 0.108
Normal bone quality, *n* (%)	97 (48)
Low bone quality, *n* (%)	106 (52)
*Laboratory variables*	
Positive CRP (≥10 mg/L), *n* (%)	88 (43)
Elevated ESR (>20 mm/Hr), *n* (%)	117 (57)
Positive RF (≥12 Ul/mL), *n* (%)	121 (60)
*Pharmacological treatment*	
Synthetic DMARDs, *n* (%)	197 (97)
Monotherapy, *n* (%)	108 (53)
Combination therapy, *n* (%)	95 (47)
Corticosteroid use, *n* (%)	147 (72)
Corticosteroid dose, mg/day: mean ± SD	4.3 ± 3.2
Time of corticosteroid use, years: mean ± SD	5.8 ± 7
Corticosteroid cumulative dose, g: mean ± SD	35.6 ± 49.9

Quantitative variables are expressed in means ± standard deviations (SDs) and qualitative variables in frequency (%). DAS28: Disease Activity Score of 28 joints, HAQ-DI: Health Assessment Questionnaire-Disability Index, DMARDs: disease-modifying antirheumatic drugs, CRP: C-reactive protein, ESR: erythrocyte sedimentation rate, RF: rheumatoid factor, TBS: trabecular bone score. Normal bone quality: TBS of 1.300 and above. Low bone quality: TBS equal to 1.299 and below.

**Table 2:** Comparison of characteristics between rheumatoid arthritis with normal vertebral trabecular bone quality (TBS ≥ 1.300) vs. low vertebral trabecular bone quality (TBS < 1.299).

Variables	RA + Normal Bone Quality *n* = 97	RA + Low Bone Quality *n* = 106	*p*
Age, years: mean ± SD	55.5 ± 10.5	61.9 ± 11.4	**<0.001**
Menopause, *n* (%)	73 (75)	96 (90)	**0.004**
Sedentary life style, *n* (%)	65 (67)	71 (67)	0.99
Hypertension, *n* (%)	33 (34)	53 (50)	**0.02**
Diabetes Mellitus, *n* (%)	4 (4)	14 (13)	**0.02**
Smoking, *n* (%)	5 (5)	16 (15)	**0.02**
Body mass index, kg/m^2^: mean ± SD	27.6 ± 4.7	27.5 ± 3.7	0.09
Bone mineral density (BMD), gr/cm^2^	1.86 ± 0.29	1.77 ± 0.31	**0.03**
Normal BMD, *n* (%)	34 (65)	18 (35)	**<0.001**
Osteopenia, *n* (%)	41 (55)	33 (45)	
Osteoporosis, *n* (%)	22 (22)	55 (52)	
Disease duration, years	12.1 ± 8	15.1 ± 11	**0.02**
Functional disability (HAQDI > 0.6)	45 (47)	41 (40)	0.31
Disease activity (DAS28-ESR ≥ 2.6)	72 (50)	73 (50)	0.52
Positive RF (≥12 Ul/mL), *n* (%)	55 (56)	66 (62)	0.42
Synthetic DMARDs, *n* (%)	96 (99)	101 (95)	0.21
Corticosteroid use, *n* (%)	73 (75)	74 (70)	0.38
Corticosteroid dose, mg/day: mean ± SD	4.6 ± 3.3	4.0 ± 3.1	0.15
Time of corticosteroid use, years: mean ± SD	6.4 ± 7.5	5.4 ± 6.4	0.30
Corticosteroid cumulative dose, g: mean ± SD	40.4 ± 57.9	31.2 ± 41	0.19

Comparison of quantitative variables (means) made with Student’s *t* test. Comparison of qualitative variables (proportions) performed with Chi-square tests. Statistical significance *p* < 0.05. TBS: trabecular bone score.

**Table 3:** Factors associated with low vertebral trabecular bone quality (TBS ≤ 1.300) in women with rheumatoid arthritis in the multivariate analysis.

Variables (*n* = 203)	Enter Method			Forward Method (Stepwise)		
	OR	95% CI	*p*	OR	95% CI	*p*
Age (years)	1.05	(1.01–1.09)	0.02	1.05	(1.02–1.08)	**<0.001**
Smoking	3.380	(1.08–10.54)	0.035	Not in the model	-	-
Menopause	1.11	(0.37–3.37)	0.85	Not in the model	-	-
Diabetes mellitus	3.20	(0.94–10.85)	0.06	3.30	(1.03–10.60)	**0.045**
Hypertension	1.38	(0.72–2.66)	0.33	Not in the model	-	-
Body mass index	0.99	(0.92–1.06)	0.78	Not in the model	-	-
Duration of RA	1.02	(0.99–1.06)	0.21	Not in the model	-	-
Functional disability	1.35	(0.69–2.66)	0.38	Not in the model	-	-
Disease activity	1.54	(0.26–1.15)	0.11	Not in the model	-	-
Corticosteroid use	1.24	(0.62–2.52)	0.54	Not in the model	-	-

Multivariate analysis: Logistic regression model. (a) Enter method; (b) Forward stepwise method. Confidence Interval. Model with dependent variable: low vertebral trabecular bone quality (TBS ≤ 1.300). Excluded covariables in forward method (stepwise): menopause, body mass index (kg/m^2^), disease duration (years), functional disability (HAQ-DI > 0.6), disease activity (DAS28-ESR > 2.6) and corticosteroid use. OR, odds ratio; 95% CI, 95% statistical significance, *p* ≤ 0.05.

## Data Availability

The datasets generated during and/or analyzed during the current study are not publicly available as they include sensitive information but are available from the corresponding author on reasonable request.
